# A Markov Model-Based Fusion Algorithm for Distorted Electronic Technology Archives

**DOI:** 10.1155/2022/4202181

**Published:** 2022-04-22

**Authors:** Lei Wang

**Affiliations:** School of Science Henan University of Technology, Zhenzhou 450000, China

## Abstract

This paper presents an in-depth study and analysis of the restoration of distorted electronic technology archives using Markov models and proposes a corresponding fusion algorithm. Using the image gradient parametrization as a regular term, the filtering restoration process is constrained and the fuzzy kernel is estimated to solve the degradation problem existing in the Tibetan antiquarian literature. In the algorithmic framework of nonlocal mean filtering, the calculation of the weight function is improved to reduce the computational effort. In the simulation results, it is shown that the improved nonlocal mean filtering restoration algorithm in this paper has good overall quality evaluation performance in the restoration of text-based images. The structural similarity between the generated image and the real image is guaranteed, and the internal mixed-mode learning of a single SAR image is performed by combining the pyramidal hierarchical network structure to improve the effectiveness of the generated image in general. The method further improves the similarity between the generated and real images, while improving the accuracy of the classification based on the data expanded by the generation method. The feasibility and accuracy of the online algorithm for the parameter estimation problem of the model are illustrated through numerical experiments with two specific examples of hidden Markov models, namely, a double Gaussian mixture model and a finite-state Markov chain model with Gaussian noise. At the same time, the advantages of the algorithm proposed in this paper are demonstrated by comparing the experimental results of the online EM algorithm with those of the offline EM algorithm on a unified model. Finally, the empirical analysis is used to illustrate the application of the algorithm in practical scenarios.

## 1. Introduction

Image restoration, as one of the important research directions in the field of image processing, covers several research areas such as graphics, stochastic processes, probability statistics, and pattern recognition and has a wide range of applications in various fields such as medical, film, and military fields. The largest one of them is chosen, and then whether a hidden state is one of the most likely hidden state sequences through the reverse pointer is determined, so that the wrong data in the hidden state sequence arrangement can be thrown away. In the case of damaged images, as the original appearance was often unclear, image restoration was mostly analyzed and executed according to the rules of human perception [1]. The use of image processing has become more widespread due to the widespread availability of the Internet and the maturity of image processing technology. However, images can be lost or damaged during transmission and storage, which affects the quality of the image and its aesthetic and usability value. Digital images play an important role in scientific research and everyday life, and image completion and image restoration are problems that are often encountered when working with digital images. Despite the challenges of implementation, image extensions will lead to many exciting and novel applications. For example, image output can be used for panorama creation, video extensions for vertical shots, and texture creation, which are of great research interest as less research has been conducted in this area [2].

Hidden Markov models have critical applications in statistics as well as in time series analysis and have had a far-reaching impact on academia and industry in the last fifty years. Classical hidden Markov models, which are hidden Markov models with a finite number of sequences, can model practical problems in a variety of scenarios. Gaussian mixture models, an important class of hidden Markov models, are a common and extremely effective modeling tool used to analyze complex models with a relatively simple structure to analyze and model a variety of complex probability density problems in real-world problems [3]. They are widely used in the field of visual media, have been theoretically studied in depth in the statistical as well as computer science communities, and have been used in numerical approximation and feature recognition; they have important applications in numerical approximation, feature recognition, classification, denoising, and reconstruction of images, as well as fault identification and diagnosis. Most of the hardware in ground-based image acquisition systems is photosensitive and is susceptible to atmospheric refraction and radiometric dispersion, which can cause degradation of the image [4]. In imaging systems in space, there is also motion blur from the speed difference between the satellite and the camera shutter and the vibration of the spacecraft. In addition to this, the interference of internal noise and external noise on remote sensing images cannot be ignored. Therefore, image restoration techniques can be used to process remote sensing images acquired from space or ground stations to restore the original face as much as possible to extract more useful information.

In terms of the mathematical principles of the Gaussian mixture model, the model is a linear combination of a set of normal distributions to form a more complex mixture model that can approximate the data distribution of a sample with multiple unknown distributions and approximate the parameters of the hidden variables from the observable samples. Moreover, according to many theoretical and numerical studies available, Gaussian mixture models can theoretically fit a wide range of smooth distributions and are therefore frequently used in practical engineering work. It is bound to bring about an impact on the result, which will cause the estimated value to be distorted. The stochastic approximation is an algorithm to reduce the influence of the error on the estimation result as much as possible, so the stochastic approximation algorithm is used to gradually approach the optimal solution. Like the hidden Markov model introduced above, the parameter estimation of the Gaussian mixture model, as a class of hidden Markov model, is also an important direction of current research, and the accurate estimation of the parameters of this model is an important prerequisite for the analysis, simulation, and solution of practical production and life problems.

## 2. Related Research

The selection of sound feature parameters for the experiments and the collection of references reveals that the researchers either combine machine learning algorithms to enhance the robustness of the extracted features or combine multiple feature parameters to form a feature parameter set [5]. The combination of multiple feature parameters extracts features on timbre and melody from the audio files and fuses these features as the initial training dataset to be trained in a Deep Belief Network (DBN) training model. The fusion of timbre and melody features is used to solve the problem that a single feature parameter in traditional music emotion classification cannot fully depict music emotion [6]. A semantic repair model is constructed using a deep convolutional generative adversarial model, which uses a priori loss and weighted semantic loss to search for the “closest” encoding of a damaged image in the hidden space, which uses a network of generators to generate the missing information [7]. The weighted semantic loss is used to determine the “closest approximation” while conditioning on the missing image and less realistic images is penalized by a priori loss.

However, in everyday life, various factors often lead to missing areas of an image, which has led to the development of digital image restoration techniques [8]. The concept was first introduced at an academic conference in 2000, where the restoration problem was transformed into a problem of solving partial differential equations through modeling. The general process of digital image restoration is to first identify the damaged area, that is, to artificially identify the damaged part of the image based on visible information such as texture, color, and structure and to mark it with a different color to distinguish the area to be restored from the good part and then to solve for the pixel-optimal problem, which transforms the known image feature information into a mathematical computational problem through mathematical modeling, and an appropriate restoration algorithm is selected to derive the damaged area from the good area [9]. Finally, the pixel filling is done by sequentially assigning the calculated pixel values to the damaged areas. Its important applications are in many fields, such as coding and compression of digital images, restoration of literary works, production of film and television special effects, and image decoding.

By using convolutional networks to constrain local texture and global content, features collected from the middle layer of the convolutional neural network are needed to generate texture and content close to the real image [10]. This can not only expand the modeling ability of the hidden Markov model but also improve the recognition accuracy of the model. The difference is that the model used in the contextual information-based approach is a traditional computational model based on mathematical formulas, whereas the CNN-based approach uses a network model generated by iterative training of large-scale data. Both methods are suitable for repairing images with relatively simple textures in the missing region, with the disadvantage that they are less effective when faced with complex textures in the background surrounding the broken region. This is because this type of method can generate new pixels with reasonable semantics based on the feature distribution of the input pixels. The disadvantage is that, due to the large oscillations and difficulty in convergence during GAN training, it is more difficult to grasp the distribution and alignment rules of the generated pixels, and the problem is that the filled region which is not consistent with the known region can easily occur.

## 3. Analysis of the Fusion Algorithm for the Distorted Electronic Technology Profile of the Markov Model

### 3.1. Markov Model Fusion Algorithm Design

The observation sequence represents the state presented by the response probability density distribution, with different states for different observation sequences [11]. The states of a hidden Markov model (HMM) are observed indirectly through the observation sequence and cannot be observed directly. This includes a hidden Markov chain with some states, where the transfer between states is random; in addition, there are a set of random functions describing the hidden correlation between observations and states, which is partly an implicit stochastic process, so the hidden Markov model is also known as a doubly stochastic process [12]. The focus will be on the probability calculation problem of the three basic problems of the hidden Markov model and its common solution algorithms. This problem is used to solve the probability of getting to the currently available sequence of observations given the parameters of the hidden Markov model, which can be expressed in mathematical terms as the posterior probability of getting to a given observation *P*(*Y|λ*) given the parameters *λ*. The common method of calculation is the forward-backward algorithm. Firstly, we introduce forward probability and backward probability, respectively, and describe their use in this algorithm.

The forward probability is the probability that, given the model parameters *λ*, the sequence of observations up to the current moment *t* is *y*_1_, *y*_2_, *y*_3_,…, *y*_*t*_, and the probability that the state at the current moment *t* corresponds to *q*_*i*_^2^ can be written in mathematical terms as

(1) $$\begin{array}{c} \alpha_{t}\left(i\right)=P\left(y_{1},y_{2},y_{3},...,y_{t},x_{t}=q_{i}^{2}|\lambda \right). \end{array}$$

By solving recursively for the forward probabilities, the posterior probability *P*(*Y|λ*) of the desired sequence of observations can be obtained, which is the basic idea of the forward algorithm. The hidden Markov model, most applied to speech recognition, was originally conceived as the idea that the human ear hears sounds from the tongue, larynx, and vocal folds and that these physiological differences in human structure work together to produce human speech. Foley sound is an important synthesis technique in sound design. The purpose of Foley sound is to create various sounds required by the plot, such as ocean waves, various friction sounds, or artificially synthesized unnatural sounds. The acquisition can also borrow the technology of Foley sound, and the newly acquired sound effect material needs to be quickly matched to the corresponding classification. The algorithmic detection system consisting of the hidden Markov model simulates the changes in the human vocal configuration as the actual hidden state, and the whole process of the change of human voice structure in the process of sound production is modeled.

The backward algorithm is like the forward algorithm in the probability that a hidden Markov model 1 with a state at moment *t* under the condition that it is and produces a partial sequence of observations from *t* to *T* is the backward probability, denoted as

(2) $$\begin{array}{c} \beta_{t}\left(i\right)=P\left(O_{t}^{2},O_{t+1}^{2},O_{t+3}^{2},...,O_{t+n}^{2}\right). \end{array}$$

Both the forward and backward algorithms are probabilities given a hidden Markov model and a set of scheduled observation sequence conditions; for different problems, one can choose hidden Markov models with different parameters triples and specific observation sequences and evaluate which hidden Markov model has the highest probability of producing a given observation sequence. In the speech recognition problem, when a series of different hidden Markov models are used, each model is modeled in the experiment corresponding to one of the individual words, and an observation sequence is formed from each pronounced word, which can be discriminated by finding the hidden Markov model with the highest probability for this observation sequence [13].

(3) $$\begin{array}{c} p^{*}=\min\delta_{T}\left(i\right). \end{array}$$

The Viterbi algorithm stores a backward pointer for each state (*t* > 1), and the local probability is the probability that the path indicated by the backward pointer reaches a state. The local probability calculation of the Viterbi algorithm is different from the local probability calculated in the forward algorithm because the probability does not change over time; the Viterbi algorithm does not count the probability of each path in the grid to reduce the computational complexity. The Viterbi algorithm calculates that the probability of reaching a certain state is the most likely path of time t and not the sum of all path probabilities. When it is not possible to find the most probable path to a state, at time *t* = *l*, the local probability of the process can be calculated by multiplying the initial probability of the state at *t* = 1 with the observation probability of the corresponding observation state, in a similar way to the forward algorithm, where the local probability is calculated by multiplying the initial probability with the corresponding observation probability.

It is very common to see “noise” in real-time dynamic data, where the probability of following the optimal path to the termination state before determining the most likely termination state is the local probability of reaching the termination state. The Viterbi algorithm will consider the complete sequence of observations, select the largest one, and then determine whether a hidden state is one of the most probable hidden state sequences utilizing a back pointer, thus throwing away erroneous data in the sequence of hidden states. The significance of this in speech processing is that, for example, distortion or loss of data for one of the phonemes in the pronunciation of a word in a training sample will not affect the recognition and classification of that word.

(4) $$\begin{array}{c} P\left(Y|\lambda \right)=\sum_{i=1}^{N}\sum_{j=1}^{N}\alpha_{t}\left(i\right)\alpha_{ij}b_{j}^{2}\left(y_{t+1}\right)\beta_{t}\left(j\right). \end{array}$$

**Figure 1:**
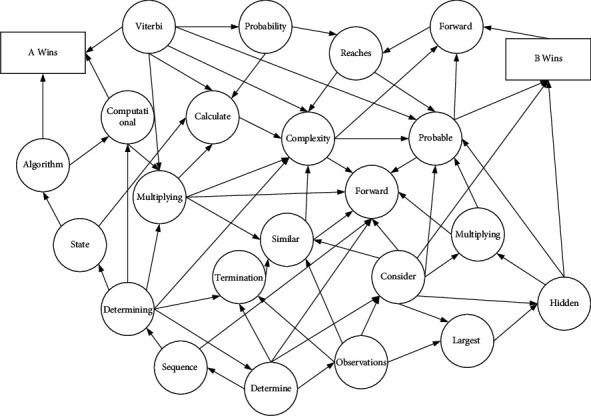
Markov model framework.

Since there are many problems in theoretical research and practical experiments that can be mathematically modeled as root problems, but for which the actual functional form is difficult to obtain directly, the zeros of the function can only be estimated from a sequence of observations with observation errors, until it is implemented only by the conv layer. Because the number of channels of the generated data is not necessarily three, in order to conform to the standard three channels of the image, both must be processed by To_RGB after operation. The stochastic approximation, however, is an algorithm created to minimize the impact of errors on the estimation results, so the stochastic approximation algorithm to gradually approach the optimal solution is an effective method to solve the problem of finding the roots of equations containing random errors, as shown in Figure 1.

Stochastic approximation was first used to solve the problem of estimating the zeros of an unknown function, that is, to solve for the roots of an unknown function that can be observed, but the value of the function that can be observed is the result after interference with the error term. Now assume that there is an unknown function *f*: *R*l ⟶ *R*2; its set of zeros is *J*={*x* : *f*(*x*)=0}. Let, at each observation point *x*_*i*_, the function *f*(*x*_*i*_) with noise be expressed as

(5) $$\begin{array}{c} y_{i+1}=f\left(x_{i}\right)-\varepsilon_{i}. \end{array}$$

Depending on the probability distribution *B* of the hidden Markov model, it can be classified into three basic forms: the continuous hidden Markov model, the discrete hidden Markov model, and the semicontinuous hidden Markov model. The three basic problems described above are all for discrete hidden Markov models, whereas, in this paper, for the case where the observed sequence is a continuous signal, a vector quantization process is required for the application, which often leads to signal distortion caused by signal quantization. To avoid such problems, the continuous hidden Markov model is chosen in this paper to build the model. As Gaussian mixture models are theoretically able to approximate arbitrary curves, they are usually used to approximate the true probability distribution of the observed sequence. This can not only expand the modeling capability of the hidden Markov Model but also improve the recognition accuracy of the model.

(6) $$\begin{array}{c} G_{MM}\left(x_{i},\Theta \right)=\sum_{k=1}^{K}c_{j}G\left(x_{i},\mu_{j},\sum_{j}\right), \end{array}$$

where the weighting coefficients need to satisfy

(7) $$\begin{array}{c} \sum_{k=1}^{K}c_{j}=0. \end{array}$$

*G* is a high-dimensional Gaussian density function with the expression

(8) $$\begin{array}{c} G\left(x^{i},\mu_{j},\sum_{j}\right)=\frac{1}{2\pi^{d/2}\sqrt{\sum }}\exp\left[-\frac{1}{2}{\left(x+\mu_{j}\right)}^{T}\sum_{j}^{-1}\left(x+\mu_{j}\right)\right]. \end{array}$$

In the process of calculating a simple Bayesian model, the probabilities of occurrence of each feature in a given classification are multiplied together and then the sum of the product terms is solved; as each product term of a multinomial is a fractional number less than 1, the successive multiplication of fractional numbers will lead to smaller and smaller values as *t* increases when the number of *t* is so large that the dynamic range of the product values exceeds the dynamic range of the product, and the program will “overflow.” To improve the quality of the generated SAR images and make their texture features have better effects on human perception, this section designs a method to add texture information constraints to the discriminator. Similarly, the backward probability does not guarantee that the result will not “overflow”; the iterative process only ensures that the computer can quickly calculate the sum of the multiplicative terms but does not solve the overflow problem [14].

**Figure 2:**
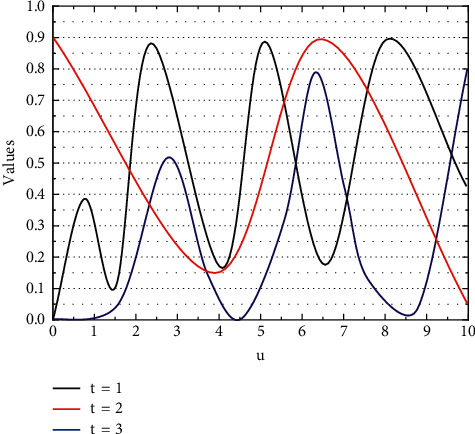
Schematic diagram of the Gaussian mixture model.

Live radio often fails to achieve the desired effect, and there are also virtual scenes where live sound effects are not available, and the only way to match the sound effect the audience wants to feel is through the technical means associated with postproduction, a process known as sound design. The purpose of sound effects is to create a variety of sounds needed for the plot, such as the sound of waves, various friction sounds, and synthetic unnatural sounds. In practice, considering the acquisition of sound effects can also borrow the techniques of sound effects; the newly acquired sound effects need to be quickly matched to the corresponding classification, so the experiments first consider the hidden Markov model training sound effects material data. The training model is shown in Figure 2.

Therefore, we take the natural logarithm of the product result in the formula, transforming the multiplication of fractional numbers into a logarithmic addition operation, which will avoid many multiplication calculations, reduce the amount of computation, remove the error caused by floating-point rounding, and ensure a high dynamic range.

### 3.2. Experimental Design for the Restoration and Fusion of Distorted Electronic Technology Archives

To steadily increase the resolution of the generated images, an incremental growth training mechanism is used. Instead of training images at the desired resolution in one step, we start by generating the smallest 4*∗*4 image and then gradually move to sharper resolutions such as 8∗8 and 16∗16 after ensuring that the current resolution has been trained consistently.

**Figure 3:**
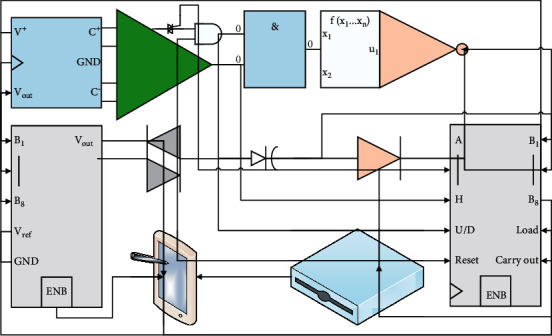
Progressive growth training mechanism.

The progressive growth training mechanism is shown in Figure 3, with 2*x* being resolution scaling via convolution and 0.5*x* being resolution reduction via pooling. The smooth transition in resolution is achieved by layer weights *α*. In the process of resolution scaling, the layer accounts for weight *α*, and the resized layer accounts for weight 1 − *α*. *α* goes from 0 to 1, indicating that initially resize acts directly, while training is intended to achieve resolution scaling through continuous learning by the conv layer until it is achieved by the conv layer only [15]. As the number of channels of the generated data is not necessarily three, both are processed by To_RGB after the operation to comply with the standard three channels of the image. The discriminator and the generator are trained in exactly the opposite way in a progressive downsampling process. For the defect parts of the image, since the original appearance is often unclear, the image restoration is mostly analyzed and executed according to human cognitive rules. The reason why this training method works so well is that it allows for better and better initialization, constantly and fully inheriting the training progress and results from the previous step and eliminating the need to relearn repeatedly, thus enhancing the stability of the model training.

This section describes the training of the restoration algorithm in detail in terms of the experimental environment, dataset, and loss function design. The effectiveness of each improvement method in improving the performance of the model and the superiority of the image restoration performance of the neural network proposed in this paper is verified through a longitudinal comparison of the model in different configurations and a cross-sectional comparison of the model with three other mainstream image restoration models, CVAE, DCGAN, and WGAN, mainly in terms of visual effects and numerical evaluation metrics.

The texture is a manifestation of image quality, which reflects comprehensive information such as brightness and greyscale variations of an image, and describes the spatial layout of an image. To humans, texture properties are a relatively intuitive image characteristic and the human eye can respond to texture, a perception that proceeds from the pixel point to the whole [16]. Therefore, to improve the quality of the generated SAR images and to make their texture features more effective to the human eye's senses, this section designs a method to add texture information constraints to the discriminator. Texture features of SAR images are an important property that describes the graphical structure in SAR images. Different targets and features have different textures, and, by improving the loss function of the generative adversarial network, it can be further constrained to learn the overall spatial features of the SAR image and obtain spatial content information.

**Figure 4:**
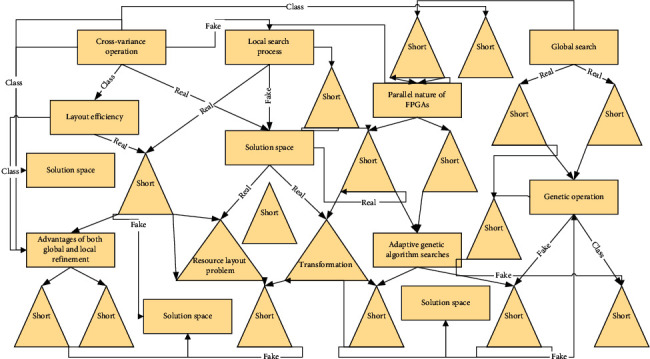
Schematic diagram of the improved experimental structure.

The LBP method first selects a central point in the image and compares it with the neighboring pixel points for the grey value and sets it to 0 if the grey difference is within the threshold, 1 if it is larger than the threshold, and −1 otherwise [17]. Image completion and image restoration are often encountered problems. Although there are quite a few challenges in the implementation process, image scaling will lead to many exciting and novel applications. According to the quantization result, the mode matrix is split into positive, negative, and equal matrices, which describe the bright, dark, and homogeneous regions in the SAR image, respectively; finally, the histograms of the three matrices are obtained separately and normalized to obtain the local mode histogram by cascading. The difference between the real SAR image and the generated image is compared using LBP, and the smaller the difference between the two is, the closer the generated image is to the texture characteristics of the real image, as shown in Figure 4.

The two-dimensional discrete wavelet of an image requires a one-dimensional discrete wavelet to be transformed to obtain it. Firstly, in the image row direction, the one-dimensional discrete wavelet transform is applied, and then the one-dimensional discrete wavelet transform is applied in the image column direction. The wavelet transform indicates the variation of the signal at various scales, also known as wavelet coefficients. After the image has undergone two one-dimensional discrete wavelet transforms, the energy in the high-frequency part is lower and most of the energy concentrated in the low-frequency part [18]. In general, it can be assumed that the wavelet coefficients with relatively large absolute amplitude have their energy provided by the real signal of the image, while the wavelet coefficients with smaller absolute amplitude are provided by external noise. The wavelet shrinkage transform method is used to obtain a smooth signal in three steps: wavelet decomposition, wavelet shrinkage, and wavelet reconstruction.

Although subjective evaluation methods are intuitive and efficient, images in nature are so varied and distorted in content that rigid adherence to the quality evaluation criteria given by the International Telecommunication Union (ITU) is not convincing [19]. In addition, subjective evaluation methods are highly random and the results can be influenced by many factors such as the knowledge of the observer. Objective quality evaluation methods for recovered images, on the other hand, do not require direct human involvement and can increase the credibility of the quality evaluation of the recovery results, and the results are stable, fast, concise, and reproducible and can be used for production industrial guidance.

There are many gaps in the acquired images which if fed directly into the recovery algorithm would increase the computational complexity and make the recovery less satisfactory [20]. This model uses a relatively simple structure to analyze various complex probability density problems in simulating practical problems. It is widely used in the field of visual media, has in-depth theoretical research in statistics and computer science, and is used in numerical approximation, feature recognition, image classification, denoising, and reconstruction, and fault identification and diagnosis have related important applications [21].

## 4. Analysis of Results

### 4.1. Markov Model Fusion Algorithm Performance Results

This section presents results for a selection of these parameter estimates. Through the iterative results of the parameter estimation for the double Gaussian mixture model, the parameters converge to the true values of the model parameters with a small number of iterations for a given initial value, and this numerical experiment verifies the feasibility of this chunked online EM algorithm in the double Gaussian mixture model scenario.

In practice, each episode of the program will inevitably have some sound effects material recorded badly in the field, resulting in unusable material in the post, and sometimes, to find replaceable material, the sound effects clips that closely resemble the target sound effects will be obtained for a particular plot context utilizing sound mimicry as replacements.

**Figure 5:**
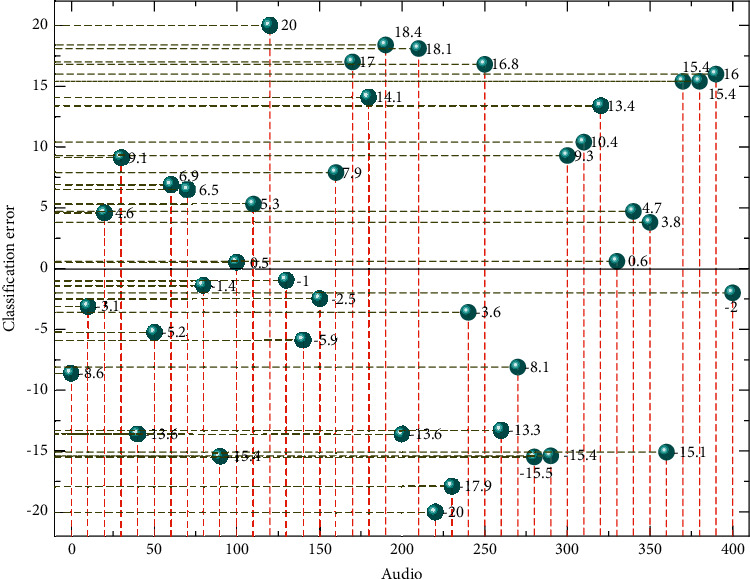
Classification error distribution for electronic audio files.

The remaining 1704 audio files (15 classifications) were selected as a new dataset to test the recognition rate of the trained back-propagation neural network. These 1704 audio files were entirely newly introduced audio files that were not in the previous feature training set, and the errors in the actual and predicted classifications of the 100th to 500th audio clips are randomly selected for observation as shown in Figure 5.

The practicality of this approach is not high, as the sound effects clips do not have the same sound features even in the same classification; for example, bicycle sounds include chain sounds or car bells, and riding on a gravel road is different from riding on a tarmac road; the same program is recorded in the same scene, and there will not be a situation where all the classified clips present new sound features. The first is model selection. For a mixed model, the unknown number of branches has always been a major difficulty in the research and analysis process. How to determine the number of branches of a mixed model is an important issue that many researchers and engineers have been paying attention to. I therefore only consider the practical requirements of the application, the use of sound mimicry to replace a categorized material at a later stage, as shown in Figure 5.

The average recognition rate of individual experimental groups, such as the replacement of street crowd sounds, sports crowd sounds, and train sounds, is not high, at about 65%. These three newly introduced sound sources contain a lot of complex sound sources, such as crowd sound, including shouting, noise, and some traffic sounds. They have high similarity, so the recognition rate is not high.

The average recognition rate of the three categories of sound effects tested was above 70%, and the average recognition rate of the three categories of sound effects tested was around 85% when the sounds of motorbikes, waves, and rivers were replaced. The average recognition rate for the group of tests where the ship sounds were replaced was 68.9%, analyzing that the newly introduced ship sounds included some wave sounds and a mixture of ship engine sounds and ship whistle sounds, which are also more complex in origin and have a higher similarity to some of the already existing categorized sound sources.

**Figure 6:**
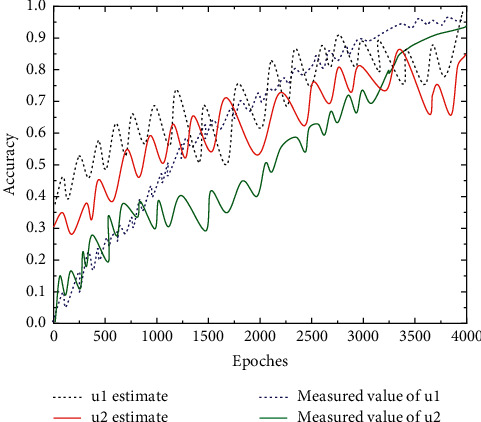
Estimation of *u*1, *u*2.

The first element of the transfer kernel, *q*_11_, with a truth value of 0.95, is given the initial values {0.7, 0.5, 0.2} for the same number of iterations as the numerical experiments described above. Similarly, for the estimation of *v*, an artificially given initial value {2, 0.8, 0.1} was used for *v* = 0.25, and the experimental results are shown in Figure 6. This experimental result amply demonstrates that the results converge to the true values of the model parameters after a sufficient and finite number of iterations for different randomly given initial values.

On the one hand, the results demonstrate the effectiveness of the online algorithm proposed in this paper, which is used to solve the parameter estimation problem of a class of hidden Markov models; on the other hand, it also effectively verifies the convergence of the algorithm from the perspective of numerical experiments; at the same time, the algorithm shows a certain degree of robustness for different initial values, as shown in Figure 6. In addition, there is a random function set that describes the hidden correlation between observations and states. This part is an implicit random process, so the hidden Markov model is also called a double random process. The chunked online EM algorithm was used for the exponential family distribution model and the double Gaussian mixture model. The chunked online EM algorithm is used in the parameter estimation problem for the exponential family distribution model and the double Gaussian mixture model.

The data used in the simulation experiments are firstly presented by simulating the sample points generated from the distributions of the exponential family distribution model as well as the Gaussian mixture model, and the online EM algorithm is used to estimate the parameters of the two models separately; furthermore, the parameters of the models are inferred from the observable samples and compared with the real parameters of the models to illustrate the feasibility of this online algorithm for the parameter estimation problem, while for the randomly assigned initial values of the parameters, it is observed that, after a finite number of iterations, the estimates of the parameters will converge to the stability point of the algorithm, also demonstrating the robustness of this online algorithm.

At the same time, by comparing the online EM algorithm with its offline counterpart in the double Gaussian mixture model, the difference is not only in the convergence of the algorithm but also because the offline EM algorithm uses the information of all samples in each iteration of the parameter update, which not only makes the traditional algorithm unsuitable for scenarios with streaming data but also causes a very large computational overhead in scenarios with very large data volumes, whereas the online algorithm can dynamically update the parameters according to the constantly updated samples, making the online algorithm efficient in parameter updates even in scenarios with large datasets.

### 4.2. Experimental Results of Distorted Electronic Technology Archive Restoration Fusion

To verify the effectiveness of the algorithm proposed in this chapter, experiments were conducted on the MOSTAR and SAR-I datasets mentioned above. For example, the MOSTAR dataset has morphological differences and diverse angles for each type of target, and the SAR-I dataset has different scattering coefficients and distributions for each type of data representing different types of features. Therefore, the adaptability of the algorithm proposed in this chapter is demonstrated by generating experiments with different categories of images from the two datasets. In the experiments, the learning effect of the algorithm and the ability to generate SAR images are demonstrated through subjective visual presentation of the generated results, evaluation of objective metrics, and evaluation of classification performance.

**Figure 7:**
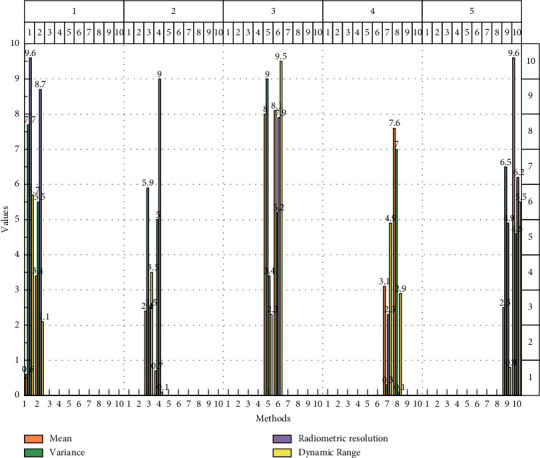
Objective evaluation results.

Two factors are considered: one is that the a priori distribution reflects the low-level characteristics of the pixel points, which may not improve the visual sensory affect much, and the other is the influence of the a priori distribution itself; as it is approximated by the traditional method, the fit is not completely accurate, so it may be biased when improving the noise potential space, as shown in Figure 7.

By comparing LBP-GAN with Method 1, it can be found that its final generated results are visually better and can have relatively clear contours, while LBP is reflecting the local greyscale variation, which can support that the proposed algorithm can better learn the spatial features of the real target, and the local binary mode approach is also helpful in capturing the local texture information of the maneuvering target. The arrangement sequence presented by these changes is used as the hidden state, and the final sound and the output sound signal are used as the observation state. The algorithm detection system is composed of the hidden Markov model, which simulates the structural changes of human vocalization as the actual hidden state. Finally, the combined distribution-based and LBP-GAN approach combines the advantages of both, and the result is closer to the real image compared to the original method.

By comparing the results of the four methods with different epochs, we can see that as the number of iterations increases, the results of all four methods slowly become more detailed from the initial noise, and the visual presentation becomes better.

It is clear from the comparison that the application of the distribution-based approach produces results that approximate the real image at an earlier epoch and that the network learns the approximate properties of the real SAR image faster, and, given that the other parameters of the network are consistent with the configuration, it can be analyzed that when the noise given to the generative network comes from a potential space that more closely matches the a priori distribution of the real image, it reflects the real image faster. The introduction of a priori information closes the distance from the initial random noise to the final true distribution to a certain extent, improving the learning speed. However, the final generation effect is not visually outstanding compared to Methods 3 and 4.

**Figure 8:**
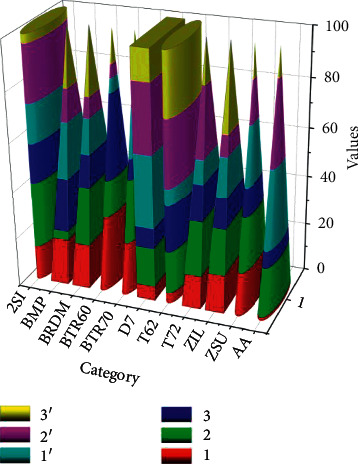
Comparison of MSTAR's distribution-based and LBP-GAN algorithms for classification experiments.

To validate the algorithms, we randomly select 200 images from the 100th to 400th epochs of each class of each algorithm for the experimental analysis. For presentation purposes, the results of ZSU234 are first analyzed separately, as shown in Figure 8.

As the amount of training data continues to increase, the classification accuracy improves for almost every category. Comparing Experiments 1 and 2, the accuracy of each category improved significantly, with the 2S1 category improving by about 29% and ZIL by about 14%. Since the probability does not change with time, the Viterbi algorithm does not count the probability of each path in each grid to reduce the computational complexity. The Viterbi algorithm calculates the probability of reaching a certain state at time *t*, the probability of the most probable path, but not the sum of all path probabilities. In comparison with Experiments 2 and 3, the amount of training data was increased again and the classification results were improved, but the improvement was lower than that of Experiment 2 compared to Experiment 1, indicating that the improvement was not significant when the training sample size was sufficient and the sample was expanded again.

The initial learning rate was 0.0002 for the discriminator and 0.001 for the generator, which were trained using the Adam optimization method. The number of model iterations was 400.

Comparing Experiments 1′, 2′, and 3′ with Experiments 1, 2, and 3, respectively, where the expanded data is derived from the images generated by the algorithm in this chapter, the accuracy improves in several categories, with 1′ improving by about 13% compared to 1 and 2S1 and BTR70 improving by about 13%. This shows that the results generated by the algorithm in this chapter can effectively learn the properties of real SAR images and help to improve the classification task.

## 5. Conclusion

Hidden Markov models are stochastic models consisting of unobservable state values and observable observations, and Markov models in which the hidden states are finite-state Markov chains are one of the important branches of this model. In this paper, the proposed online EM algorithm is used to calculate a Gaussian mixture model. In this paper, we demonstrate the feasibility and effectiveness of the proposed online EM algorithm by numerical simulations and experiments on exponential family distribution models and finite Gaussian mixture models. The proposed online EM algorithm is extended to the chunked data scenario, where the chunked online algorithm can update the parameters in batches, enabling the convergence of the parameters with fewer iterations than the common online EM algorithm. The paper also successfully improves the training speed of the model by using residual blocks, as well as solving the possible degradation problem by adding mean square error and Markov random field energy function to the training loss in conjunction with the GAN loss to train the model and avoid the blurring problem of the generated images. The experiments demonstrate that the quality of the generated images and the training speed of the model significantly improved after the improvement of the model and the loss function.

## Data Availability

The data used to support the findings of this study are available from the corresponding author upon request.
